# Etude du faible poids de naissance associé à l’âge maternel et la parité dans une population couple mère-enfant suivi à Lubumbashi

**DOI:** 10.11604/pamj.2015.20.246.5169

**Published:** 2015-03-16

**Authors:** Prosper Kakudji Luhete, Olivier Mukuku, Prosper Kalenga Muenze Kayamba

**Affiliations:** 1 Département de Gynécologie-Obstétrique, Faculté de Médecine, Université de Lubumbashi, République Démocratique du Congo

**Keywords:** Faible poids de naissance, parité, âge maternel, Lubumbashi, low birth weight, parity, maternal age, Lubumbashi

## Abstract

**Introduction:**

Evaluer l'influence de l’âge maternel et de la parité sur la naissance d'un faible poids de naissance (FPN) à Lubumbashi.

**Méthodes:**

Il s'agit d'une étude basée sur une analyse documentaire des dossiers médicaux des accouchées enregistrées dans les maternités des 10 hôpitaux généraux de référence (HGR) de la ville de Lubumbashi en République Démocratique du Congo entre le 1^er^ décembre 2013 et le 31 mars 2014. Ces accouchées ont été réparties en deux groupes, en fonction du poids de naissance de leurs enfants: groupe I (femmes ayant accouché de nouveau-nés vivants dont le poids était inférieur à 2500 grammes) et groupe II (femmes ayant accouché de nouveau-nés vivants dont le poids était supérieur ou égal à 2500 grammes). Il s'agissait dans tous les cas de grossesses monofoetales âgées de 28 semaines ou plus. L’âge maternel et la parité ont été étudiés et comparés dans les deux groupes. Les données ont été analysées à l'aide des logiciels Épi info version 7.0 et SPSS version 19. Les différences étaient jugées significatives pour un seuil p < 0,05.

**Résultats:**

La prévalence du FPN chez les nouveau-nés issus de ces accouchées était ainsi de 6,4% (71/1112). En analyse univariée, les femmes d’âge < 20 ans présentent un risque multiplié par 2,47 fois d'avoir un nouveau-né de FPN comparativement à celles de ≥20 ans (OR = 2,47; IC95%: 1,26-4,78) et celui pour les primipares d'avoir un enfant de FPN est 2,3 fois supérieur à celui de multipares (OR = 2,32; IC95%: 1,34-3,99). En faisant la régression logistique, nous avons observé que seule la parité est significativement corrélée au poids de naissance (OR ajusté = 2,61; IC95%: 1,43-4,74).

**Conclusion:**

L’étude que nous avons menée montre que le taux de FPN diminue considérablement avec l’âge de la mère à partir de 20 ans et la multiparité.

## Introduction

Le taux de mortalité néonatale est un indicateur majeur de la qualité des soins obstétricaux et néonataux [1]. Le quatrième objectif du millénaire visant à réduire la mortalité infantile de 2/3 d'ici 2015 passe par une réduction de la mortalité néonatale [2]. Depuis longtemps, le faible poids à la naissance sert d'indicateur de la santé périnatale en raison de son lien avec la survie, l’état de santé et le développement du nouveau-né [3]. Dans le monde, le faible poids de naissance (FPN) représente la principale cause de mortalité périnatale et infantile [4] et environ 24% de 4 millions de décès néonatals enregistrés chaque année dans le monde sont dus aux complications liées à la prématurité [5]. En 2008, à Lubumbashi (RDCongo), une étude cas-témoins réalisée à la maternité de l'hôpital général de référence Jason Sendwe trouve un risque de décès périnatal de près de 16 fois chez les FPN (OR ajusté = 15,7; IC 95% = 11,2-22,0) [6]. Outre cette surmortalité, il y a beaucoup de preuves suggérant que les FPN soient associés aux risques pour la santé tant au moment de la naissance que plus tard dans la vie [7, 8]. Les deux principales causes d'insuffisance de poids à la naissance sont l'accouchement prématuré (avant que 37 semaines de grossesse soient terminées) et le retard de croissance intra-utérin [9, 10].

L'influence de l´âge maternel et la parité sur le poids de naissance a déjà été étudié dans le passé par de nombreux chercheurs en Afrique comme dans le monde. Mais quant à ce qui concerne la RDCongo, il n'existe que peu de travaux publiés sur ce sujet; c'est le cas d'une étude menée dans la province du Maniema par Kyamusugulwa sur le FPN dans les milieux ruraux [11] et d'une autre menée à Kamina (province du Katanga) par Kangulu sur les facteurs de risque de FPN [12]. C'est ainsi que nous avons mené cette étude qui s'est fixé comme objectif d’évaluer l'influence de l’âge maternel et de la parité sur la naissance d'un FPN à Lubumbashi.

## Méthodes

Il s´agit d´une étude basée sur une analyse documentaire des dossiers médicaux des accouchées enregistrées dans les maternités des 10 hôpitaux généraux de référence (HGR) de la ville de Lubumbashi en République Démocratique du Congo (hôpital militaire de Ruashi, Cliniques Universitaires, hôpital Jason Sendwe, HGR Katuba, HGR Kenya, HGR Kamalondo, HGR Kisanga, HGR Kampemba, hôpital Gécamines-Sud et hôpital SNCC) entre le 1er décembre 2013 et le 31 mars 2014. Ces hôpitaux sont répartis dans les 7 communes que compte la ville de Lubumbashi. Ces femmes ont été réparties en deux groupes, en fonction du poids de leurs enfants: un premier groupe (groupe I), qui comprenait les femmes qui avaient accouché de nouveau-nés vivants dont le poids était inférieur à 2500 grammes (FPN) et un second groupe (groupe II), composé de femmes ayant accouché de nouveau-nés vivants dont le poids était supérieur ou égal à 2500 grammes. Il s´agissait dans tous les cas de grossesses monofoetales âgées de 28 semaines ou plus.

Les données qui ont été recueillies et répertoriées sur une fiche individuelle établie pour chaque parturiente provenaient des dossiers obstétricaux établis lors de l´admission en salle d´accouchement. L’âge maternel et la parité ont été étudiés et comparés dans les deux groupes. Les données ont été analysées à l´aide des logiciels Épi info version 7.0 et SPSS version 19. Nous avons utilisé le test de khi2 pour la comparaison des proportions, et le test de Student pour celle des moyennes. Les différences étaient jugées significatives pour un seuil p< 0,05. Par ailleurs, nous avons effectué une analyse par régression logistique, en ayant adopté la procédure descendante pour le choix du modèle final pour une signification statistique au seuil de 5%. La naissance d'un nouveau-né de FPN (< 2500 grammes) est considérée comme variable dépendante de notre étude et les variables indépendantes sont l’âge maternel (< 20 ans et ≥ 20 ans) et la parité (primiparité et multiparité (parité≥ 2)).

## Résultats

Entre le 1^er^ décembre 2013 et le 31 mars 2014, 1203 accouchements ont été enregistrés dans les 10 maternités de la ville. Nous avons analysé tous les dossiers des accouchées et parmi celles-ci, 1112 avaient une gestation unique dont 71 faisaient partie du groupe I et 1041 appartenaient au groupe II. La prévalence du FPN chez les nouveau-nés issus de ces accouchées était ainsi de 6,4% (71/1112). L’âge moyen des mères d'enfants avec FPN est de 26,6±6,6 ans allant de 14 à 44 ans alors qu'il est de 28,6±6,1 ans allant de 14 à 46 ans chez celles d'enfants normopondérés; la comparaison de ces deux moyennes donne une différence statistiquement significative (t = 2,66; p = 0,0077).

Le Tableau 1 rapporte la répartition de l’âge maternel en fonction de groupes. Treize pourcent des accouchées âgées de moins de 20 ans ont eu des enfants de FPN contre 5,7% de celles dont l’âge était supérieur ou égale à 20 ans; il ressort que l’âge maternel est en association significative avec le poids de naissance (p = 0,0062) et les femmes d’âge < 20 ans présentent un risque multiplié par 2,47 fois d'avoir un nouveau-né de FPN comparativement à celles de ≥ 20 ans (OR = 2,47; IC95%: 1,26-4,78). En ce qui concerne le poids de naissance en fonction l’âge maternel, la moyenne pondérale chez les accouchées âgées de moins de 20 ans est de 3105,0±555,8 grammes allant de 1400 à 4400 grammes alors qu'elle est de 3222,9±518,4 grammes allant de 750 à 5250 grammes chez les accouchées d'au moins 20 ans. La comparaison de ces deux moyennes donne une différence statistiquement significative (t = 2,23; p = 0,0258) (Tableau 1).

**Tableau 1:** Distribution des accouchées selon leur âge et en fonction du poids des nouveau-nés

Age maternel	PN < 2500 g		PN ≥ 2500 g		Total		P	OR (IC95%)
	n	%	n	%	n	%		
<20 ans	14	13,0	94	87,0	108	100	0,0062	2,47 (1,26-4,78)
≥20 ans	57	5,7	947	94,3	1004	100	-	1
**Total**	71	6,4	1041	93,6	1112	100		

La parité moyenne est de 2,8±2,2 variant entre 1 et 10 chez les mères d'enfants avec FPN alors qu'elle est de 3,9±2,5 variant entre 1 et 15 chez celles d'enfants normopondérés; en comparant ces deux moyennes, l'analyse statistique montre une différence significative (t = 3,71; p = 0,0002).

Des 212 primipares, 24 d'entre elles (soit 11,3%) ont eu des enfants de FPN contre 5,2% de multipares; l'analyse statistique montre qu'il existe une association significative entre la parité et le poids de naissance (p = 0,0018) et que le risque pour les primipares d'avoir un enfant de FPN est 2,3 fois supérieur à celui de multipares (OR = 2,32; IC95%: 1,34-3,99) (Tableau 2).

**Tableau 2:** Distribution des accouchées selon la parité et en fonction du poids de naissance des enfants

Parité	PN < 2500 g		PN ≥ 2500 g		Total		p	OR (IC95%)
	n	%	n	%	n	%		
1	24	11,3	188	88,7	212	100	0,0018	2,32 (1,34-3,99)
≥2	47	5,2	853	94,8	900	100	-	1
**Total**	71	6,4	1041	93,6	1112	100		

Quant à la moyenne du poids de naissance des enfants en fonction de la parité de leur mère, elle est de 3093,9±522,7 grammes chez les enfants des primipares variant entre 1450 et 4500 grammes alors qu'elle est de 3239,2±519,6 grammes variant entre 750 et 5250 grammes chez ceux des multipares; l'analyse statistique montre une différence significative entre ces deux moyennes (t = 3,66; p = 0,0003).

Quant à la régression logistique, la variable dépendante de notre étude est la survenue de FPN. Les variables indépendantes sont: l’âge maternel et la parité. Nous avons effectué une régression logistique multiple. Celle-ci a permis la construction d'un modèle comprenant l’âge maternel et la parité. Nous avons observé que la parité est significativement corrélée au poids de naissance en ajustant sur la variable âge maternel (Tableau 3).

**Tableau 3:** Analyse par régression logistique

Variable	OR ajouté	IC95%	Z-Statistic	P-Value
Age maternel	1,05	0,98-1,12	1,55	0,1202
Parité	2,61	1,43-4,74	3,14	0,0017

## Discussion

Le poids de naissance est maintenant largement utilisé comme un indicateur de l´état de santé des individus et des populations car il a des fortes associations avec la santé des enfants et des adultes. Le FPN est associé au retard de croissance physique, au déficit cognitif et au handicap [13].

Il est classiquement rapporté que les caractéristiques maternelles et les événements survenant en cours de grossesse ont un impact sur le poids de l'enfant à la naissance [14–16]. L´effet de l´âge de la mère sur le poids de naissance de l'enfant a été un sujet de débat. Certaines études signalent que les mères adolescentes sont plus susceptibles de donner naissance à des enfants de FPN mais d´autres suggèrent que d´autres facteurs tels que les différences raciales et socio-économiques peuvent confondre ces résultats et affaiblir des conclusions quant à l´effet de l´âge maternel [17–20]. Cette influence de l´âge de la mère sur le poids de naissance de l'enfant avait déjà été rapportée dans plusieurs études [11, 14, 16, 21]. En analyse univariée, notre étude rapporte une proportion de 13% des accouchées âgées de moins de 20 ans ayant eu des FPN contre 5,7% de celles dont l’âge était ≥ 20 ans (OR = 2,47; IC95%: 1,26-4,78; p = 0,0062) signifiant que plus la mère est jeune (<20 ans), plus le poids de naissance est faible et elle court le risque de 2,47 de donner naissance à un enfant de FPN. Les études antérieurement menées par Karim à Dhaka(Bangladesh), Feleke à Addis Ababa (Ethiopie), Chukwudi à Enugu (Nigeria) et Muula au Malawi avaient également trouvé des proportions élevées d'enfants de FPN chez les mères de moins de 20 ans [22–25]. Bisai, dans son étude menée à Kolkata (Inde), rapporte que près de 44% des mères âgées de moins de 20 ans ont donné naissance à un enfant de FPN [26].

Par ailleurs, l’étude réalisée par Kyamusugulwa au Maniema (République Démocratique du Congo) montre que l’âge de la mère est un facteur qui influence le FPN, particulièrement chez une jeune mère de 14 à 17 ans et cela dans les deux maternités étudiées [11]. D'autres auteurs retrouvent un âge de ≤16 ans comme âge maternel à risque de mettre au monde un FPN qui est plus bas qu'au nôtre [27, 28]. Cependant, à l'hôpital de Ngozi au Burundi, de 2001 à 2003, Nkurunziza constate que les mères âgées de 25 ans et moins avaient un risque d'accoucher des enfants de faible poids de naissance deux fois supérieur à celui de mères de plus de 25 ans [29]. Dans une étude menée par Fraser, après exclusion de certains paramètres confondants, il s'est avéré que le jeune âge demeure un facteur de risque de FPN et de naissance prématurée lors de la première naissance [30]. Mukhopadhyay, dans une étude observationnelle transversale réalisée dans l´Est de l´Inde, a enregistré une proportion élevée d'enfants de FPN chez les primigestes adolescentes comparativement aux primigestes adultes [31]. Les explications proposées pour cette prédominance d'enfants de FPN chez les mères adolescentes seraient biologiques, c'est-à-dire qu'une adolescente est en pleine croissance et une fois enceinte elle se retrouve en compétition pour les nutriments avec le foetus qui se développe ou que la grossesse, dans les deux ans suivant la ménarche, augmente le risque d´accouchement prématuré et afin, à cet âge, il a été noté une faible efficacité des fonctions placentaires [32, 33]. Hormis cet aspect biologique, il existe aussi des facteurs socio-psychologiques qui peuvent également être impliqués car chez les adolescentes, beaucoup de grossesses ne sont pas planifiées, sont non désirées ou découvertes tardivement. En plus, les adolescentes qui deviennent mères manquent d´expériences, sont plus susceptibles que d´autres d´être pauvres, d´être sous-scolarisées ou de vivre dans des zones à accès limité aux ressources et aux services des soins de santé [14, 32, 34–37]. C'est ainsi qu'en 1992, le gouvernement indien avait recommandé que la naissance du premier enfant ne devrait pas avoir lieu avant 20 ans d´âge maternel [38].

Par contre, quelques résultats publiés par certains auteurs contredisent notre constat; c'est ainsi que les mères d’âge compris entre 20 et 30 ans et celles >30 ans sont retrouvés comme à risque de donner naissance à des enfants de FPN respectivement par Bhatnagar et Sharma [39, 40]. Quant à Beddek, l’âge maternel associé au FPN est celui allant de 20 à 34 ans [41]. Ces divergences notées par ces auteurs en rapport avec nos résultats pourraient s'expliquer par les critères de recrutement utilisés. En effet, certains auteurs comme nous, avaient recensé toutes les grossesses monofoetales âgées de 28 à 42 SA après exclusion des morts foetales in utero et des malformés congénitaux [21, 26], mais pour d'autres auteurs, toutes les grossesses avaient été incluses sans tenir compte du nombre du contenu utérin [41] et d'autres encore ne se sont limités qu’à recruter uniquement les grossesses âgées d´au moins 37 SA [42].

Nous avons trouvé une relation significative entre la parité et le poids de naissance. Nous avons noté que les nouveau-nés des primipares pesaient moins que ceux des multipares avec une différence pondérale moyenne de près de 150 grammes statistiquement significative en défaveur des primipares (p = 0,0003). Nos résultats d'analyse multivariée affirment cette forte association entre la primiparité et le FPN avec un rapport des côtes ajusté de 2,61 (p = 0,0017) et montrent que le poids de naissance augmente avec la parité, mais la relation entre le poids de naissance et l´âge de la mère est plus faible que la précédente. Nos résultats sont en cohérence avec ceux de plusieurs auteurs qui mettent en évidence un risque de FPN fortement associé à la primiparité [43–46]. Plusieurs auteurs africains ont le même constat; c'est le cas de Beddek, à Sidi Bel Abbes (Ouest de l'Algérie), qui note que la proportion des nouveau-nés de FPN est la plus élevée chez les primipares avec une différence statistiquement significative en comparaison avec les autres tranches de parité (p < 0,0001) [41]. De même à Yaoundé (Cameroun), Chiabi identifie la primiparité comme étant un facteur de risque de survenue du FPN [47]. Nkurunziza trouve que le risque de mettre au monde des enfants de FPN est 4,7 fois chez les primipares que chez les multipares [29]. Au Malawi, Muula fait aussi la même constatation [25].

Cependant ceci n'est pas seulement observé dans les pays africains mais il s'agit d'une observation aussi bien pour les pays en développement que les pays développés. Au Vietnam, Vaktskjold trouve une association positive entre la parité et le poids de naissance indépendamment des autres facteurs de risque de FPN étudiés [48]. En 2006, l’étude menée en Inde par Bisai avait observé une différence significative de poids moyen à la naissance de 158 grammes entre les nouveau-nés des mères primipares et ceux des multipares en faveur de ces derniers [26].

Aux Etats-Unis, dans une étude recherchant l'influence de la parité sur le FPN menée sur 36 056 nouveau-nés singletons newyorkais constate que le poids de naissance augmente de façon similaire lorsque la parité va de 1 à 3, mais diminue nettement dans les groupes de parité plus élevée [49]; et dans une méta-analyse faite par Shah, la primiparité était associée au FPN avec un risque de 1,41 (1,26-1,58) [50]. En Caroline du Nord, Swamy conclut que la parité exerce une plus grande influence sur le poids de naissance que l’âge maternel, avec des effets significativement différents à travers les sous-groupes raciaux [21].

## Conclusion

L’étude que nous avons menée montre que le taux de FPN (faible poids de naissance) diminue considérablement avec l’âge de la mère à partir de 20 ans et la multiparité. Le fait que la grossesse chez les adolescentes qui sont majoritairement primipares est à risque élevé de donner naissance à des bébés de FPN justifie la sensibilisation communautaire pour la prévention des grossesses chez des primipares (à la fois dans le mariage ou hors mariage), sensibilisation qui pourrait, en terme de santé publique, réduire considérablement la proportion d'enfants de FPN et donc permettre d’éviter d'autres conséquences chez les nourrissons nés avec un poids <2500 grammes.
